# A systematic review of sacral nerve stimulation for faecal incontinence following ileal pouch anal anastomosis

**DOI:** 10.1007/s13304-017-0496-y

**Published:** 2017-10-30

**Authors:** E. Kong, S. Nikolaou, S. Qiu, G. Pellino, P. Tekkis, C. Kontovounisios

**Affiliations:** 1 Department of Colorectal Surgery, Chelsea and Westminster Hospital, London, UK; 2 Department of Surgery and Cancer, Imperial College, 369 Fulham Rd, London, SW10 9NH UK

**Keywords:** Ileal pouch anal anastomosis, Restorative proctocolectomy, Sacral nerve stimulation, Sacral neuromodulation, Faecal incontinence

## Abstract

Faecal incontinence is a common complication of ileal pouch anal anastomosis (IPAA) and seems to worsen with time. The aim of this paper is to review the evidence of the use of sacral nerve stimulation (SNS) for patients with faecal incontinence after IPAA. A literature search was performed on PubMed and Cochrane databases for all relevant articles. All studies, which reported the outcome of SNS in patients with faecal incontinence after IPAA, were reviewed. Three papers were identified, including a case report, cohort study and retrospective study. The total number of patients was 12. The follow-up duration included 3 months, 6 months and 24 months. After peripheral nerve evaluation, definitive implantation was performed in 10 (83.3%) patients. All three studies reported positive outcomes, with CCF scores and incontinence episodes improving significantly. Preliminary results suggest good outcome after permanent SNS implant. Studies with larger sample sizes, well-defined patient characteristics and standardized outcome measures are required to fully investigate the effect of SNS in IPAA patients.

## Introduction

Ileal pouch anal anastomosis (IPAA), first described by Parks and Nicholls in 1978 [1], is the procedure of choice for patients with inflammatory bowel disease (IBD) and familial adenomatous polyposis (FAP) requiring surgery [2].

The overall functional outcome of IPAA is generally excellent [3–6]. However, faecal incontinence is a common complication of IPAA and seems to worsen with time [4]. Faecal incontinence is defined as the involuntary passage of rectal contents (faeces, flatus) through the anus and the inability to delay bowel movement until it is convenient. The issue should last at least 1 month and occurs in a child older than 4 years old and who has previously attained continence.

At 12 months post-IPAA, it has been reported that 19% of patients suffered occasional daytime incontinence and 49% suffered nocturnal incontinence [7]. Consequently, this can have a significantly negative impact on the quality of life of patients [8, 9].

Neuromodulation has gained support over the past 18 years as a treatment for faecal incontinence without any significant damage to the anal internal and external sphincters. By delivering chronic low-voltage electrical stimulation to the sacral spinal nerves, the muscles of the anal sphincter are recruited. The most established of neuromodulation treatments is sacral nerve stimulation (SNS) [10].

In practical terms, SNS involves the implantation of a programmable nerve stimulator in the subcutaneous tissue under general anaesthesia without the use of muscle relaxants. This delivers a continuous low amplitude electrical stimulation through the sacral nerve which can be accessed via the S3 or S4 foramen [11].

It has been shown that 80% of patients undergoing SNS with faecal incontinence not responsive to medical therapy had a > 50% improvement in symptoms [10]. Long-term results have shown a successful maintenance rate of 71% in patients 10 years after permanent SNS implant, with 50% maintaining full continence [12].

This systematic review will look at the use of SNS on faecal incontinence in IPAA patients.

## Method

A literature search was performed on PubMed and Cochrane databases for all relevant articles. The following keywords were used in various combinations to conduct the search: ‘sacral nerve stimulation’, ‘SNS’, ‘restorative proctocolectomy (OR coloproctectomy)’, ‘pouch’, ‘IAA’ and ‘IPAA’. All studies which were identified in this search were analysed for relevance and content.

## Results

**Fig. 1:**
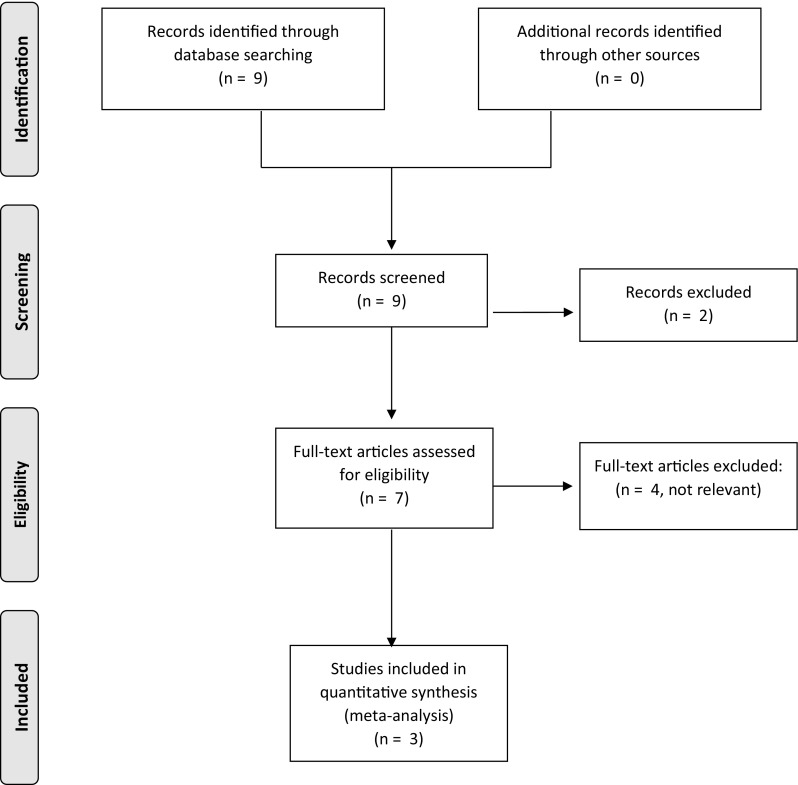
PRISMA flow diagram

Nine studies were identified in the initial search and reviewed. Two studies were excluded based on the abstract alone. Four studies assessed the electrophysiology of the puborectalis muscle using SNS and not the long-term effect of SNS on faecal continence and therefore were deemed irrelevant to this review by both authors. The remaining three studies included 12 patients [11, 13, 14]. The characteristics of these studies are shown in Table 1 (Fig. 1).

**Table 1:** Characteristics of the studies

Study	Design	Data	No. of patients	Age	Sex	Indication
Meurette et al. [13]	Case study	ND	1	46	M	Severe attack of acute colitis
Lebas et al. [14]	Cohort study	April 2012–May 2013	4	Mean: 57 (22–60)	4F	Ulcerative colitis: 2/4 Crohn’s: ¼ FAP: ¼
Mege et al. [11]	Retrospective study	January 2006–December 2014	7	ND	ND	ND

In 2010, Meurette et al. [13] was the first to report the outcome of SNS for faecal incontinence following an IPAA in a case report. Meurette et al. reported the stimulation of the right S3 nerve root in a 46-year-old male patient who was referred for a severe attack of acute colitis. A subtotal colectomy with end ileostomy was performed and then 3 months later, a completion proctectomy with an IPAA reconstruction and a defunctioning loop ileostomy was performed, which was later reversed. He suffered faecal incontinence after the initial surgery for 3 years which had not improved despite optimised medical therapy and biofeedback. Endonanal ultrasonography showed no sphincter disruption. He experienced up to eight bowel movements per day with nocturnal soiling, with a Wexner/Cleveland Clinic (CCF) Incontinence Score of 16. An improvement, greater than 75% in incontinence episodes was experienced after percutaneous nerve evaluation (PNE) was performed on the right S3 nerve root. A permanent SNS device was then implanted. His CCF score had improved to less than 4 and frequency of stools per day had decreased to 5 with no nocturnal soiling at 1 year post-implantation.

Lebas et al. [14] described four female patients who had undergone SNS for severe faecal incontinence following restorative proctocolectomy and IPAA despite optimised medical therapy. The median age was 57 years (range 22–60 years). Patients had suffered faecal incontinence for a median duration of 54 months (range 20–160 months) prior to this study. Three of the four patients had a successful response to the PNE stimulation trial and continued to receive a permanent SNS implant. At 6 months after SNS, the median number of faecal incontinence episodes per week decreased from 4 (pre-SNS) to 0.5 (post-SNS). The median frequency of stools decreased from 8 (pre-SNS) to 5 (post SNS). Their ability to defer defecation had also improved from 6 min (pre-SNS) to 90 min (post-SNS). CCF scores also improved from 15 (pre-SNS) to 7 (post-SNS). Quality of life assessment for the three patients had improved in all categories at 3 and 6 months post-SNS. The patient who underwent IPAA for FAP reported perfect faecal continence 1 month post-SNS (Tables 2, 3).

**Table 2:** Treatment details of PNE and permanent SNS implant

Study	No. of patients	No. undergoing SNS	Anaesthesia type	SNS lead	Test period	Indication for permanent implant	Follow-up duration
Meurette et al. [13]	1	1	ND	ND	3 weeks	>75% improvement in incontinence episodes	2 years
Lebas et al. [14]	4	3	GA	Quadripolar electrode	20 days	≥50% reduction in the number of FI episodes per week and/or ≥ 50% reduction in the number of FI days per week	6 months
Mege et al. [11]	7	6	ND	Quadripolar electrode	3 weeks	≥50% reduction in the number of FI episodes per week and/or ≥ 50% reduction in the number of FI days per week	3 months

**Table 3:** Results of the Cleveland Clinic Score and change in the median number of faecal incontinence episodes per week, before and after permanent SNS

Study	No. of patients	Duration of FI before SNS	Median no. FI episodes/week		Median daily stool frequency		Wexner cleveland clinic (CCF) score	
			Pre	Post	Pre	Post	Pre	Post
Meurette et al. [13]	1	3 years	ND	ND	8 with nocturnal soiling	5 without nocturnal soiling	16	<4
Lebas et al. [14]	4	54 months (range 20–160 months)	4 (4–25)	1.1 (0–4)	8 (5–12)	5 (4–6)	14.5 (13–15)	5.7 (0–10)
Mege et al. [11]	7	ND	4 (2–9)	1.8 (0–3.5)	11 (7–12)	5 (4–6)	15 (7–19)	1.5 (0–14)

Mege et al. [11] assessed the effectiveness of SNS on patients with faecal incontinence following colorectal resections, which included proctocolectomy with IPAA, rectal resection and left hemicolectomy. 7 out of 16 patients had undergone restorative proctocolectomy. Data for these seven patients were extracted from this study for analysis. A stimulation test was performed on the patients. Six of the seven patients had a satisfactory response to the SNS stimulation test and underwent pulse generator implantation for SNS. At a median follow-up of 18 (3.5–91) months, the median number of faecal incontinence episodes per week decreased from 4 (pre-SNS) to 1.8 (post-SNS). The CCF score decreased from 15 to 1.5. Daily stool frequency had also improved from 11 to 5 stools per day.

## Discussion

This systematic review demonstrates the outcome of 10 patients who had undergone SNS.

All three studies reported positive outcomes, with CCF scores and incontinence episodes improving significantly. These results are promising, as they demonstrate the effect of SNS when other optimised medical therapies had been exhausted, and therefore improving the quality of life of patients. However, these results should be interpreted with caution. There are many confounding factors which can affect the results which include patient demographics such as age, gender, pre-existing bowel function and indication for IPAA. In addition to this, the pathophysiology of anorectal incontinence is not fully understood. As a result, it is still difficult to correlate subjective and objective parameters to predict outcome for each patient and hence determine which patients would benefit most from current treatment modalities. Current scoring systems including the most commonly used Wexner incontinence score, is based on subjective assessment of severity and frequency and does not include any physiologic test parameters which may have an effect on the result of SNS use [15].

It is also important to consider the shape of the pouch and its effect on faecal incontinence. In this review, not all articles commented on the type of pouch they had or whether they had tried any other treatment before SNS or in combination with it except a brief mention of failed optimised medical management. A Cochrane review in 2012 suggested that using SNS with pelvic floor muscles may confer some benefit although due to weakness of the data this is also not certain [16].

Risk factors implicated in faecal incontinence in patients who had an IPAA include advancing age at the time of surgery, longer disease duration preoperatively, being female and having lower preoperative maximum anal squeeze pressure which can affect the results of the SNS treatment [17].

Overall, there is a lack of good evidence in the literature to support the use of SNS in patients with faecal incontinence complications following IPAA. Studies with a larger sample size and longer follow-up duration are required to reciprocate these results. There is inconsistency in the literature regarding long-term SNS outcome on faecal incontinence. Some studies demonstrated a significant sustained long-term improvement in symptoms [18] whilst others reported a loss in long term efficacy for unknown reasons [19]. Future studies with longer follow-up durations investigating the outcome of SNS in IPAA patients should take this into consideration. This review may help plan larger randomised prospective studies in the effect of SNS on faecal incontinence following IPAA.

The mechanisms of action of SNS are unknown. However, studies have illustrated using electrophysiological assessments that sacral nerve terminal motor latency [20, 21] and anal resting and squeezing pressures are markedly improved after SNS [22, 23]. Continence is the result of both the anal sphincter functional contraction and the integrity and coordinated function for the surrounding pelvic muscles. SNS is hypothesized to affect both, and therefore its effect is multifactorial.

## Conclusion

The evidence to support the use of SNS for faecal incontinence after IPAA remains very limited. Studies with larger sample sizes, well-defined patient characteristics and standardized outcome measures are required to investigate the effect of SNS in IPAA patients fully.
